# A Study of Nature’s Method for the Cure of Asiatic Cholera

**Published:** 1903-04

**Authors:** W. B. M’Laughlin

**Affiliations:** Austin, Texas


For Texas Medical Journal.

### A Study of Nature's Method for the Cure of Asiatic Cholera.

BY W. B. M'LAUGHLIN, M. D., AUSTIN, TEXAS.

In approaching this subject, it may be well to review a few of the facts, admitted to be true by the majority of observers, which refer to the etiology and course of this disease.

First. That the comma bacillus, discovered by Koch in 1883, is a specific organism, intimately connected with, and causing Asiatic cholera is, I believe, now generally admitted.

Second. That the comma bacillus is an organism extremely sensitive to heat, growing best at temperature of from 30° to 40° C. (86° to 104° F.); that its growth is interfered with at temperatures from 40° to 42°C. (104° to 106°F.) and is absolutely inhibited at the latter temperature.

Third. That an attack of cholera does not confer an immunity to a subsequent attack.

That cholera is a disease accompanied, as a rule, by vomiting, purging and a rise of from 2° to 5° of the internal bodily temperature, and that it terminates in one of three ways: By direct convalesence; by a febrile reaction, or by death from collapse.

Admitting the above observations as true, it seems to me that the conservative efforts of nature are directed to ridding the organism of the vibrio, by the vomiting and purging, and if the infection is so severe that she cannot accomplish the object by this alone, she raises the bodily temperature to a point when the growth of the vibrio is interfered with, or even inhibited. Unfortunately, both the diarrhea and the fever are a severe tax on the vitality of the patient, and he frequently succumbs from collapse, or the febrile process, once started, refuses to be controlled, and he dies from hyperpyrexia.

In most of the infectious or contagious diseases, it is now believed that they are limited as to the time of their duration, by the formation of an antitoxine which inhibits their growth, and the best efforts of many modern investigators have been directed toward the isolation of these antitoxines, so that, by their administration, we might bring about an artificial crisis in the disease, many days in advance of the time required by nature to accomplish the same object. In this manner the patient is saved days of suffering and danger.

In my opinion, heat in cholera is the analogue of Behring's antitoxin in diphtheria, and that by causing an early rise in temperature in cholera patients, we can render the disease one of an extremely low mortality.

Remembering the experiments of Martin and Han, in which, by giving an enema, to a healthy adult, of water at a temperature of 115°, the temperature of the person was raised 5 degrees in 25 minutes, I determined to adopt this method of raising the body temperature on account of the auxiliary advantage in flushing out the colon thoroughly. I used a normal salt solution of

112°, and passed from one to two quarts above the sigmoid flexure. In all I used this treatment on five cases.

The first case was in a state of extreme collapse at the time the injection was given, and died within the hour without any sign of reaction.

The second case died within ten hours with hyperpyrexia.

After this the temperature was reduced to 110°, and an ice bag was applied to the patient's head during its administration. The three remaining cases made good convalesence, entering the stage of reaction, in each case, within an hour of the administration of the injection.

I am aware that these observations are incomplete, and imperfect, but illness compelled me to leave the Philippines at this time, and I had no opportunity to give this method further trial. I therefore submit this article as it is, hoping that the ideas outlined herein may be of value in the treatment of this terrible disease.

